# Hospital Concerts and Yorkshire Wakes

**Published:** 1911-09-09

**Authors:** Alfred Whitham

**Affiliations:** Secretary Beckett Hospital Saturday and Sunday Fund Movement.


*To the Editor of* The Hospital.

### HOSPITAL CONCERTS AND YORKSHIRE WAKES.

*To the Editor of* The Hospital.

Sir,—After reading the interesting notes in your issue of August 19 re hospital concerts in Germany, it might be of use to know what music is doing for our hospital at Barnsley.

It is the custom in Yorkshire for each village to hold what is called the "annual feast," or "wakes," once a year, when friends and relations gather together to enjoy each other's hospitality. This custom was taken advantage of some years ago by musical enthusiasts for the benefit of Beckett Hospital, Barnsley, who established at each out-township within the influence of the hospital annual musical festivals. By these means during the past twenty-eight years £7,352 has been realised, of which sum £437 was raised during last year. Already during the present year some thirty successful "sings" have taken place. Each village each year appoints its own committee, who are responsible for carrying out the project on their feast Sunday afternoon. The local musicians are assisted by a permanent orchestra, and vocal party from the central committee at Barnsley. All the offerings at the festivals are voluntary, and when the weather is favourable very successful gatherings are held. You will find from the enclosed programme the system adopted at each service. I am somewhat surprised that these means of raising money are not more generally adopted.

Yours faithfully,

Alfred Whitham,

Secretary Beckett Hospital Saturday and Sunday Fund Movement.

